# Effect of the Radial Velocity Distribution on the Loss Generation of a Contra-Rotating Fan in a Ventilation System

**DOI:** 10.3390/e25030433

**Published:** 2023-03-01

**Authors:** Xingyu Jia, Xi Zhang, Kui Guo, Xuehui Li

**Affiliations:** 1 School of Mechanical Electronic & Information Engineering, China University of Mining & Technology-Beijing, Ding No.11 Xueyuan Road, Haidian District, Beijing 100083, China; 2 China North Vehicle Research Institute, Beijing 100072, China

**Keywords:** radial velocity distribution, contra-rotating fan, entropy production, numerical simulation

## Abstract

Quantification of the loss generation of ducted contra-rotating fan (CRF) blades is difficult to achieve, since there are no guide vanes between rotors. A blade design program was established to investigate the relationship between radial velocity distribution and incurred loss. Numerical and experimental techniques were used to confirm the optimal configuration’s overall performance. The relationship between loss and velocity distribution under the impact of spanwise load distribution was confirmed by the entropy contour from various perspectives. The appropriate radial velocity distribution can improve the operating efficiency of a CRF by reducing the entropy around the annulus under design and near-stall conditions. This regularity could provide some strategies in the design of contra-rotating blades.

## 1. Introduction

A contra-rotating fan (CRF) has a unique configuration compared to traditional fans. There are no guide vanes between its front and rear rotor, and its contra-rotating rear rotor (RR) cancels out a large proportion of the swirl resulting from its front rotor (FR). Therefore, the kinetic energy of the swirl can be recovered and utilized directly. In response to increasingly challenging global energy and environmental problems, this compact configuration has developed in many areas, such as high bypass ratio engines [1], unmanned aerial vehicles [2], and ventilation systems [3], due to its high power-to-weight ratio.

Using a CRF, the flow can be propelled further out of the outlet than traditional fans. However, wake interactions and periodic effects are more obvious, and cause considerable loss generation. In addition, the increase in degrees of freedom brings a significant challenge for loss-reducing designs.

In recent years, there have been very few research reports on reducing the losses of subsonic CRFs. Inspired by the wing angle structure of large migratory birds, Gao et al. [4] used bionic methods to improve the blades’ aerodynamic performance, reducing the generation of shedding vortices. Their wing angle structure divided the blade into upper and lower parts and caused the airflow passing through the upper half of the blade to flow toward the blade tip, which destroyed the conditions for generating the tail-shedding vortex. Their wing angle blade tip generated smaller entropy production with decreased velocity components. Therefore, the total pressure efficiency was increased by 7.24% at the design condition and by 11.32% on average under the entire flow condition. At the same time, the total pressure increased by 1.76% and 3.88%, respectively. Tuhin et al. [5] followed the degree of reaction formula to redesign their counter-rotating fan blade shape. The swirl resulting from its front rotor is completely canceled out by its rear rotor. They investigated four ratios of aerodynamic loading—(1) 50–50%, (2) 55–45%, (3) 60–40%, and (4) 65–35%—in the front and rear rotor, respectively. They found that increasing the loading in the FR would result in a tip losses increment and consequent axial gap losses. Because additional flow blockage was created in the tip area, the flow in the rest area accelerated more than was desired, which caused the RR blade to operate at the off-design condition and led to higher vortex-induced losses.

For single-stage blades, to improve the performance of a low-pressure axial flow fan, Ding et al. [6] adopted bending and twisting laws constructed by Bézier curves. After the optimization with a non-dominated sorting genetic algorithm, the spanwise load distribution of the fan blade became more uniform and led to a less adverse pressure gradient. Therefore, the corner separation at the trailing edge was inhibited, which increased its maximum efficiency by 5.44%. Pan et al. [7] introduced a spatially non-uniform dihedral design method to reduce losses of NASA Stage 67 by about 7.7%. The design strategy was implemented by modifying its stacking line. Generated blade force in the radial direction near the end wall drove the low-momentum fluids near the blade tip to move toward the mid-span section. Thus, this dihedral design method could smooth velocity variation in the radial direction without affecting its tangential velocity component. In addition, the flow separation intensity was weakened by reducing the blade load near the trailing edge. Chuang et al. [8] enhanced the flow capacity of a fan by optimizing its stacking line, which could increase the blade’s average axial velocity along the spanwise direction and reduce the tip leakage flow loss by reducing the tip leakage vortex intensity, which weakened its influence on the downstream area. Adjei et al. [9] improved the performance of an axial flow fan by adjusting blade parameters, such as twist, sweep, and hub thickness distribution. They found that optimized velocity distribution could improve the separation distribution and enhance flow stability, which reduced flow losses at the hub and shroud areas. Kim et al. [10] examined the effect of an airfoil’s maximum thickness position on an axial fan’s aerodynamic performance. They summarized that the highest performance could be expected due to narrowed loss region near the hub when the maximum thickness position was at 30% of the chord length. They concluded by summarizing the effect of maximum thickness position on the blade loading and velocity distribution. The resulting change in the incidence angle near the blade tip influenced the trajectory and loss generation of the tip leakage, recirculation, and backflow.

To sum up, the essence of loss reduction is to realize the appropriate load and velocity distributions achieved from a better blade shape. However, there is still no clear path to finding a suitable distribution for CRF blades. There is also little research on the relationship between radial velocity distribution and the loss generation for CRFs.

In this article, a CRF in a ventilation system was studied to improve its operating efficiency. One objective of this study is to find an appropriate radial velocity distribution form for a CRF. Another objective is to figure out the loss generation mechanism of contra-rotating blades in terms of their radial velocity distributions. Consequently, a blade design program mentioned in the abstract was established to investigate the relationship between the radial velocity distribution of contra-rotating blades and their loss generation. This work provides some advice on the design strategy for contra-rotating blades from the perspective of loss generation. The research procedure can be divided into four parts. The primary content includes the design method, numerical and experimental test techniques, verifications, and regularity analysis.

All abbreviations and subscripts are listed in Abbreviations part.

## 2. Design Method and Variable Scope

As mentioned above, the overall performance of a contra-rotating fan (CRF) mainly depends on the axial spacing between the blades and the load distributions [11,12]. Therefore, the blade design program was established to generate CRF blades by setting the axial spacing and blade profile to investigate the relationship between aerodynamic parameters and loss generation. The design specifications are listed in Table 1. The basic geometric parameters of the channel and blades were decided upon by the national standard of the People’s Republic of China, which regulates the basic types, sizes, parameters, and characteristic curves of fans [13]. For the convenience of production, this standard also requires the same motor specifications for both FR and RR of a CRF, resulting in the same nominal speed. In the present article, the load allocation ratio (FR/RR) equal to one helps to reduce the research variables and eliminate the effect of load allocation at a rotor scale.

**Figure 1:**
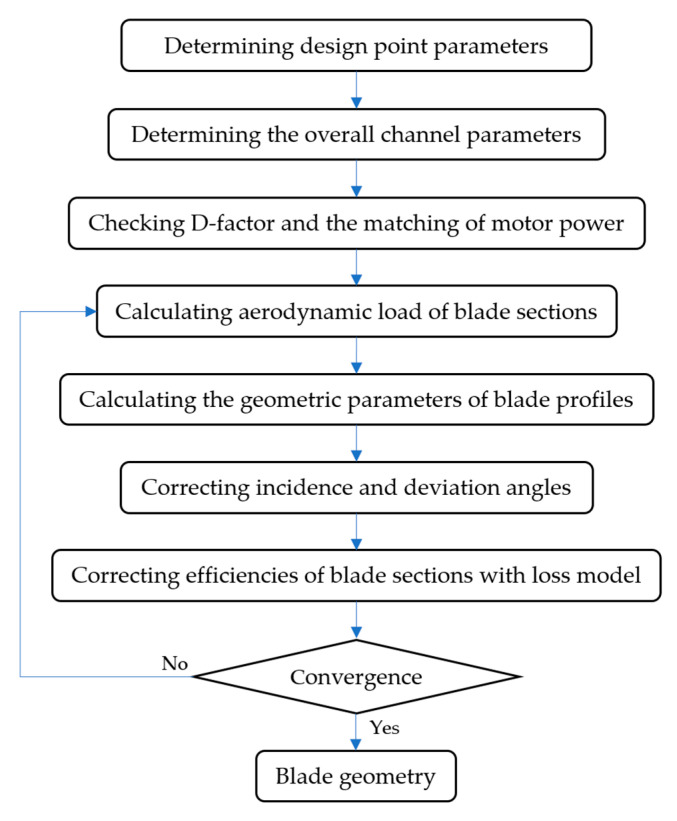
Main steps in the CRF blade design program.

In this blade design program, S1/S2 stream surface theory [14] simplifies and decomposes complex three-dimensional flow into two types of three-dimensional flow surface: S1 (blade-to-blade surface) and S2 (meridional surface). In addition, axisymmetric and incompressible assumptions [15] were also adopted to simplify the flow. The NACA-65 series profiles were utilized in this phase. According to corrected incidence and deviation angle data [16], these profiles perform well in low subsonic compressors. An end wall losses model (containing secondary flow losses) established by Howell [17] was cited and embedded in the program. After several iterations, the final blade geometry can be received. The main steps of the program are summarized in Figure 1.

### 2.1. Axial Spacing

**Figure 2:**
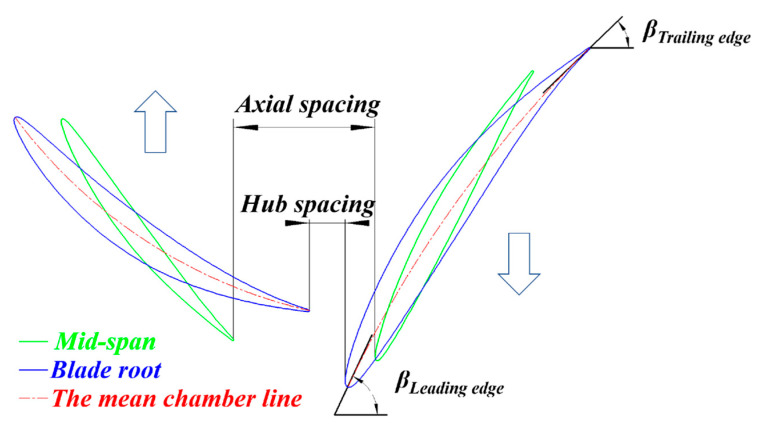
Definition sketch used for axial spacing.

In this blade design program, length ratio *x* is expressed by Equation (1) to describe the axial spacing that is illustrated in Figure 2.

(1) $$axial\;spacing=x(l_{FR}+l_{RR})\;\;\;\;\;\;\;\;x\in \left[0.5,\;1\right]$$

where *l* is the length of the mean chamber line at the mid-span of blades, and subscripts stand for different rotors.

The lower boundary of *x* is 0.5. If *x* is smaller than 0.5, the FR trailing edge (TE) is close to the RR leading edge (LE). The upper boundary of *x* is 1 because the efficiency of CRFs starts to drop when *x* is around 0.8, which will be discussed later.

### 2.2. Blade Profile

The blade profile was determined by the load distribution, which also affects the radial velocity distribution and loss generation. In this article, the load distribution of the FR is expressed by Equation (2). To maintain total pressure as a constant along the RR blade’s TE, the RR’s total pressure rise distributions are complementary to the FR’s.

(2) $$P_{t,\;\;FR}=\frac{Constant_{P}}{r^{a}}\;\;P_{t,\;\;RR}=P_{t,\;\;ES}-P_{t,\;\;FR}$$

**Figure 3:**
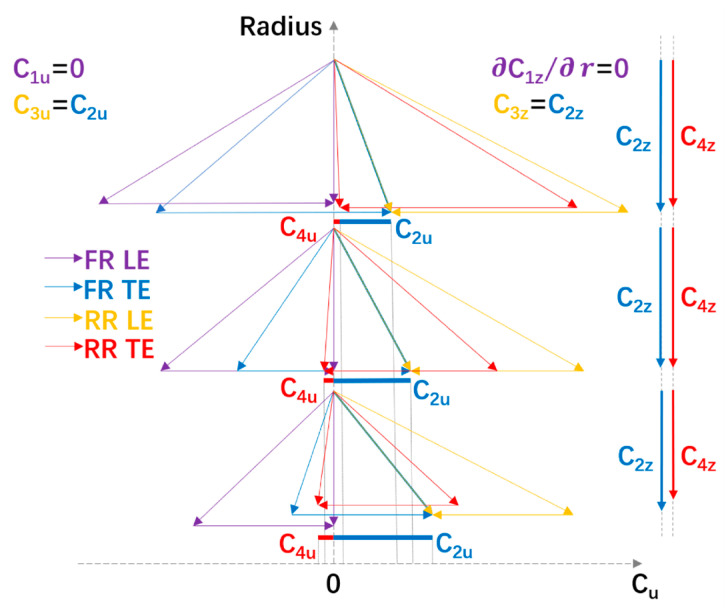
Definition sketch of ideal velocity triangles at the mid-span, hub, and tip sections (when *a* < 1).

By solving the Euler turbine equation,

(3) $$\frac{\partial P_{t}}{\partial r}=\rho \frac{\partial \left(\Delta C_{u}\right)}{\partial r}$$

the variation in $\Delta C_{u}=\left(\Delta C_{u,TE}-\Delta C_{u,LE}\right)$ can be calculated along the radius. Ideal velocity triangles along the radius are shown in Figure 3.

Each triangle includes velocity components near the LE and TE of the front and RR blades. It is apparent in Figure 3 that $C_{2u}$ reduces with an increasing radius while $C_{4u}$ rises (from a negative value to a positive value).

**Figure 4:**
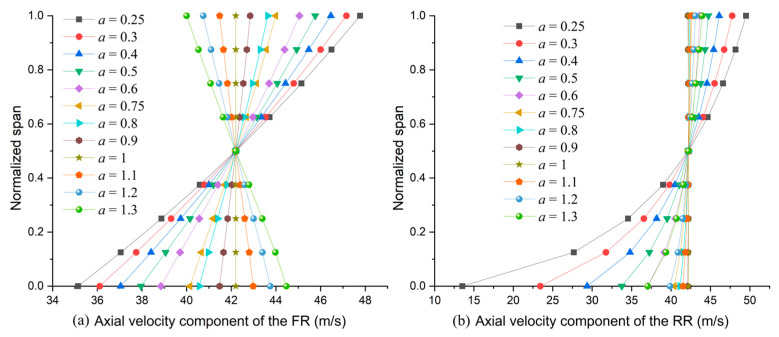
Axial velocity component distributions at the (**a**) FR TE and (**b**) RR TE.

According to the balance equation,

(4) $$\frac{1}{\rho }\frac{\partial P_{t}}{\partial r}=\frac{1}{2}\;\left[\;\frac{1}{r^{2}}\;\frac{\partial {\left(rC_{u}\right)}^{2}}{\partial r}+\frac{\partial C_{z}^{2}}{\partial r}\;\right]$$

axial velocity distributions with different exponents near the FR TE and RR TE are illustrated in Figure 4a,b. Twelve pairs of velocity distribution values were calculated to generate blade profiles for subsequent loss investigation. When *a* < 1, axial velocity (the axial component of absolute velocity), $C_{2z}$, and $C_{4z}$ increase along the span. Moreover, $C_{2z}$ is faster than $C_{4z}$ around both shroud and hub. On the other hand, when *a* > 1, $C_{2z}$ decreases along the span while $C_{4z}$ is still increasing.

It is worth pointing out that the exponents’ lower and upper boundaries are 0.25 and 1.3, respectively. Outside of the boundary, the blade profile at the RR blade root starts to behave like profiles in a turbine with negative turning angles, as listed in Table 2.

## 3. Simulation, Experiment, and Verification

Both numerical and experimental techniques confirmed the effectiveness and accuracy of the mentioned blade design program. The numerical technique was also utilized to generate sufficient data for subsequent regularity analysis. The following are details of these test techniques.

### 3.1. Numerical Technique

The numerical simulations were executed using the ANSYS-CFX solver after dividing the control volumes into structured grids in the TURBOGRID module. An eddy viscosity model, the k-ω-based SST (shear stress transport) turbulence model [18], was selected for steady-state simulation (Reynolds-averaged Navier–Stokes [19]). This turbulence model combines the advantages of the k-ε model (good at predicting high Reynolds number flow in the outer region) and the k-ω model (good at predicting low Reynolds number flow in the viscous sublayer and buffer layer) by using two functions, blending function F1 as a multiplier and blending function F2 as a viscosity limiter, to formulate the eddy viscosity [18], which could have a better prediction of the shear stress in the boundary layer. To meet the requirements for the k-ω model to predict the velocity distribution of a low Reynolds number flow in the viscous sublayer, which obeys the logarithmic law of the wall, the first element offset parameter near the wall, y+, was set as 1. The resulting settings were saved as a template in the WORKBENCH platform to ensure operational consistency in different simulation cases.

**Figure 5:**
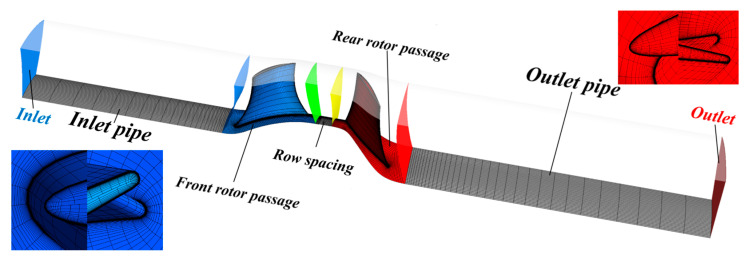
Control volume layout.

The flow medium was the ideal gas. The control volume was divided into five parts: inlet pipe, FR passage, row spacing, RR passage, and outlet pipe, as shown in Figure 5. Frozen rotor interfaces connected the parts to deal with the multiple frames of reference. Atmospheric (atm) parameters were 298.15 K and 1 atm. The flow was normal to the boundary at the inlet. The outlet mass flow rate was set to determine a specific operational condition.

The grid sensitivity analysis experiment was performed on the validation case (*a* = 0.5, *x* = 0.8), and the results are listed in Table 3. The total pressure rise and efficiency calculated by the third and fourth groups of grids were similar and tended to be stable, so the third group was adopted here.

### 3.2. Experimental Technique

**Figure 6:**
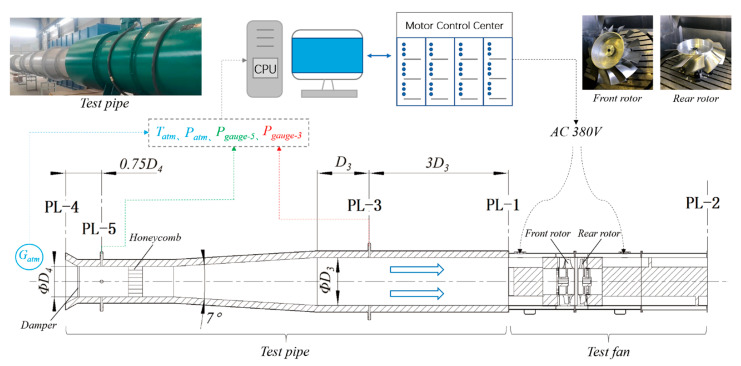
Test facility layout.

The test rig was built according to ISO-5801 standards (category C) [20]. Figure 6 shows its layout and testing fundamentals. $D_{3}$ equals the tip diameter of the CRF. A motor control center drives two three-phase asynchronous motors (2950 rpm, 30 kW). Since both motors connected to a rotor directly, their outpower was calculated through line voltage and current. The efficiency of the motor is 92.7%, and its power factor equals 0.89. A honeycomb was placed downstream of plane-5 (PL-5) to homogenize the incoming flow through the conical mouth and damper (adjusting mass flow rate). In each plane, static gauge pressure was collected from four evenly distributed measuring holes on the test pipe, and the weighted average value was finally adopted. They were measured using SETRA 268 and scaled at ±5 kPa, with an accuracy of ±1.0%. Atmospheric temperature (*T_atm_*) was measured using THT-N263A (maximum range up to 50 °C) at Plane 4, with an accuracy of ±1.0%. Atmospheric pressure (*P_atm_*) was measured using SETRA 276 (ranging from 80 to 110 kPa) with an accuracy of ±0.25%. All of the accuracy meets the requirements of ISO-5801 standards.

According to standards, the mass flow rate, *q_m_* (kg/s), was calculated according to the pressure difference between the atmosphere and Plane 5,

$$q_{m}=f\left(P_{atm},T_{atm},P_{gauge-5},D_{4}\right)$$

The test fan’s total pressure rise, *P_t_* (Pa), was calculated according to the pressure difference between the atmosphere and Plane 3. $N_{e}$ (kW) is motor power;

$$P_{t}=f\left(P_{atm},T_{atm},P_{gauge-3},P_{gauge-5},D_{3},D_{4},N_{e}\right)$$

Therefore, total pressure efficiency is

$$\eta_{Test}=\frac{q_{m}P_{t}}{\rho_{m}N_{r}}$$

where $\;\rho_{m}\;$(kg/m^3^) is the average density between Planes 1 and 2. $N_{r}\;$(kW) is rotor power.

### 3.3. Velocity Distribution Verification

**Figure 7:**
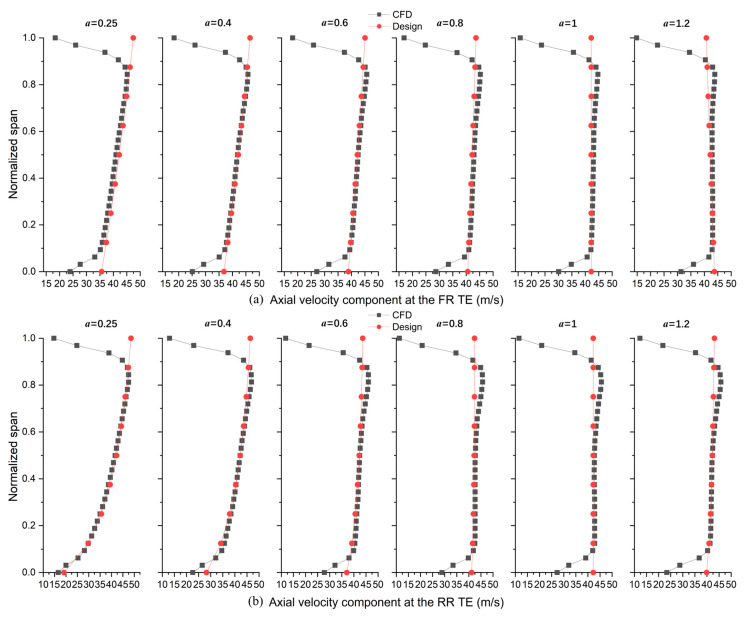
Comparison of axial velocity component distributions against CFD results (**a**) at the FR TE and (**b**) at the RR TE.

The accuracy of the design program was confirmed by the axial velocity component distributions at the FR and RR TE (*x* = 0.8). In Figure 7, CFD results fit well with the expected distribution in Figure 4a,b. However, because of frictions on the annulus walls (no-slip wall in the boundary), the CFD results deviate from the design values around the hub (below 10% span) and shroud (above 85% span). At tip clearance, the main flow cannot receive the energy from the blade directly, together with the interaction with leakage flow, which leads to the main flow’s velocities (axial component) decreasing more around the shroud than the hub. Moreover, the velocity of the main flow around the shroud decreases further when it passes the RR shroud.

### 3.4. Overall Performance Verification

The accuracy of the simulation technique was confirmed by testing the overall aerodynamic performance of the test case (*a* = 0.5, *x* = 0.8) made of the 7075 aluminum alloy. Blade profile data are listed in Table 4.

The test results at the design point are compared in Table 5. The simulation error of total pressure rise against its design value is 2.6%, and the error of efficiency against its design value is 0.7%. In addition, because of flow friction around the annulus and leakage flow through the tip clearance [21], there is a 4.5% loss in flow capacity compared to the designed mass flow rate.

**Figure 8:**
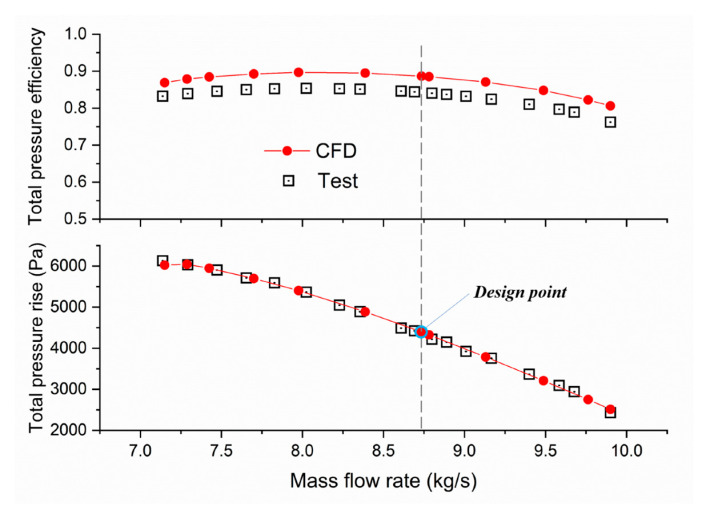
Comparison of CFD results against test data.

The comparison of overall performance is shown in Figure 8. The tendency of the simulation results fits well with the test data. The test pipe (category C) could ensure the test accuracy of total pressure at Plane 1 but not at Plane 2 due to the ignorance of the rotational kinetic energy at the outlet. Therefore, the tested total pressure at Plane 2 is slightly smaller than the simulation results (around 3.5%), which also leads to underestimating the real total pressure efficiency. Moreover, because of flow losses from supporting rods, radiator covers of motors, and rotor spacing (necessary for counter-rotating), the max test error of efficiency is around 4.8%.

The tendency of the simulation results fits well with test data, which means the above numerical technique can be utilized to generate data sets for the subsequent regularity analysis.

## 4. Regularity Analysis

Using the design program mentioned in Section 2, 72 pairs of CRF blades were built with different exponents and length ratios, listed in Table 6, for regularity analysis. Their aerodynamic performances at the design point were obtained using the numerical technique mentioned in Section 3.

### 4.1. Efficiency Analysis

**Figure 9:**
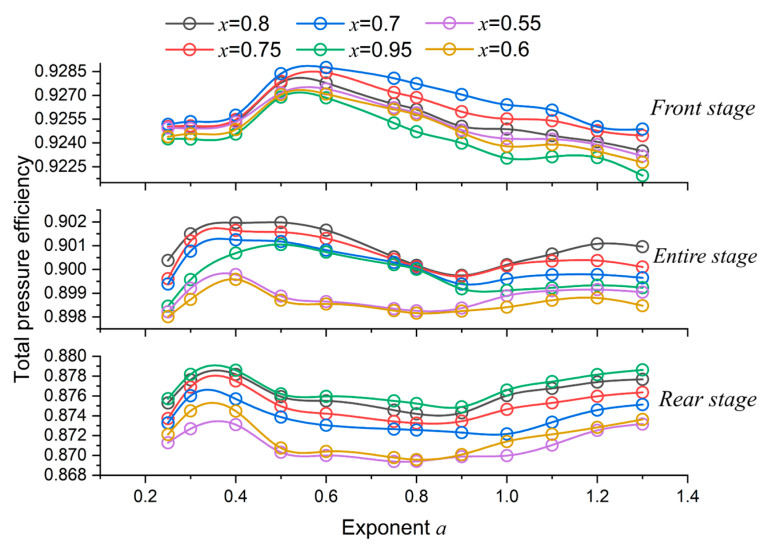
Effect of exponent and length ratio on total pressure efficiency.

The tendencies of stage efficiency (the stage refers to control volumes from the FR passage to the RR passage in Figure 5) versus exponents are illustrated in Figure 9. For different length ratios, the tendencies are similar: the maximum efficiency is located at around *a* = 0.5 for the front stage and around *a* = 0.3 or 1.3 for the rear stage. The entire stage’s maximum value is situated at around *a* = 0.4–0.5.

**Figure 10:**
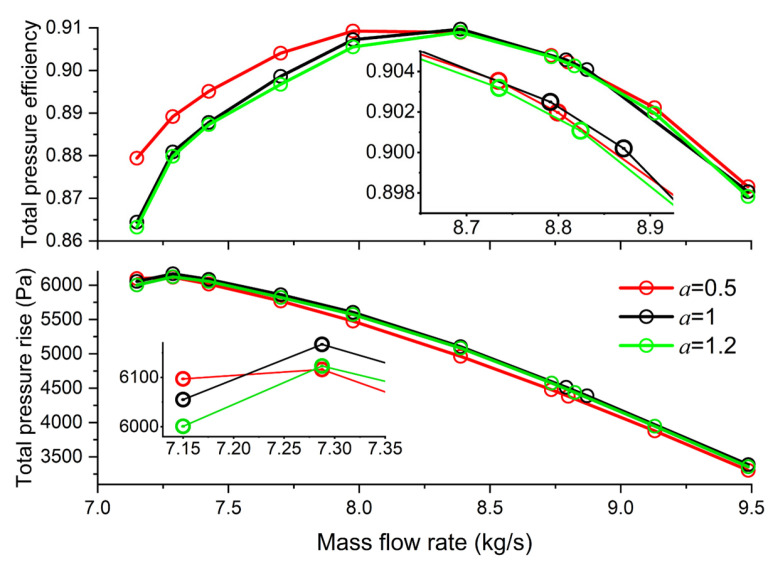
Effect of exponent on overall performance (*x* = 0.8).

Furthermore, Figure 10 presents the effect of the exponent on overall performance. For comparison with the case design via the free vortex model, cases were selected at conditions where the exponents were 0.5 and 1.2.

It is evident that when the exponent equals 0.5, the entire stage’s efficiency is the highest across almost the whole operational state with a tiny work drop. Improvements are undeniable near the stall point. The efficiency elevates by 1.5% compared to the case designed under the free vortex model (*a* = 1).

According to the first and second laws of thermodynamics, the increment of entropy can be expressed as

(5) $$ds=\frac{dh}{T}-\frac{dp}{T\rho }$$

As Equation (5) suggests, in a compression system the enthalpy could not only turn into a useful form, an increase in pressure, but also a harmful form, entropy production. In this article, the main cause of entropy production is viscous and turbulent dissipation due to the shear strain of the interaction between wake, vortex structures, and the annulus boundary layer. These flow phenomena determine the flow losses.

**Figure 11:**
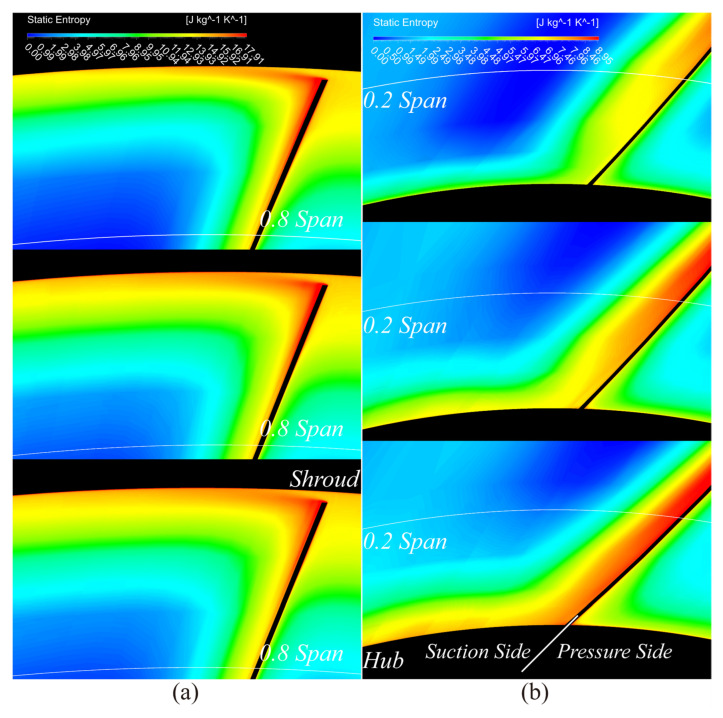
Comparison of entropy production at the RR TE near the stall state. (From top to bottom: a = 0.5, 1, and 1.2, respectively); (**a**) above 80% of the blade span and (**b**) below 20% of the blade span.

As shown in Figure 11a,b, the improvements are confirmed at the RR TE. Above 80% of the blade span, the high entropy fluid released from the blade tip is slightly weakened. Below 20% of the blade span, entropy production is significantly reduced at the boundary layer of both annulus and suction surface walls.

### 4.2. Loss Analysis

For comparison of the loss generated around blades, the spanwise integral of the relative total pressure loss coefficient is defined in Equation (6),

(6) $$I_{rel,\;loss}=\int_{0}^{1.0\;span}\frac{2(P_{rel,LE}-P_{rel,TE})}{\rho_{LE}W_{LE}^{2}}dL\;P_{rel}=P_{s}+0.5\rho \left[W^{2}-{\left(\omega r\right)}^{2}\right]$$

**Figure 12:**
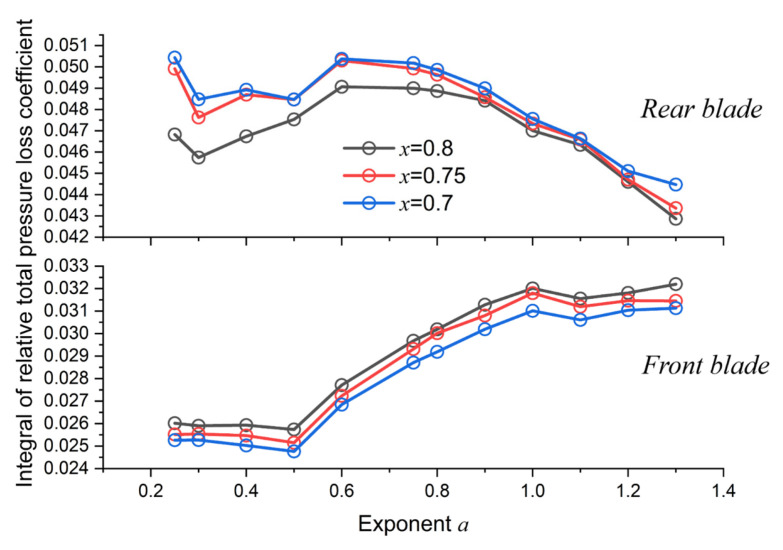
Effect of exponent on the spanwise integral of losses.

As shown in Figure 12, the tendencies of the accumulated loss for both blades coincide with the efficiency curves in Figure 9. The investigated cases are where the length ratio equals 0.7, 0.75, and 0.8, respectively, because the quasi-extreme value is located near these cases.

To interpret loss variations, spanwise loss distributions were analyzed by zooming in on the cases where *a* = 0.3, 0.75, and 1.2 (around the quasi-extreme value) when *x* = 0.8.

**Figure 13:**
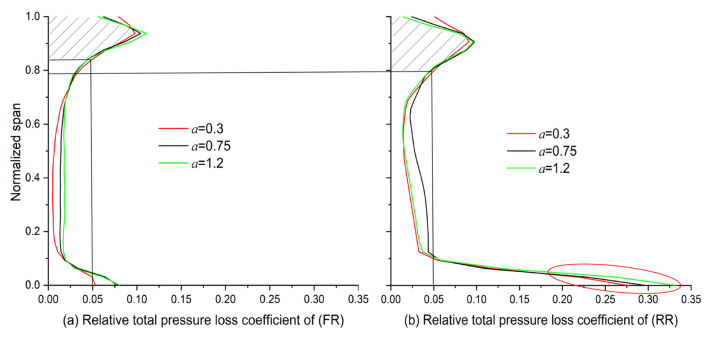
Effect of exponent on the loss of (**a**) the FR and (**b**) the RR along the spanwise direction (*x* = 0.8).

As illustrated in Figure 13, when the exponent is around 0.3, the generated loss is the smallest along almost the whole FR and RR blade span, except above 93% of the blade span. This phenomenon will be interpreted later in the analysis of entropy production. Compared with the loss generated in the FR blade channel in Figure 13a, the loss area increased slightly after the main flow passed through the RR blade tip in Figure 13b. In contrast, because of the further development of the boundary layer and the interaction with the secondary flow around the RR blade root (below 10% of the blade span), the generated loss increased drastically here.

**Figure 14:**
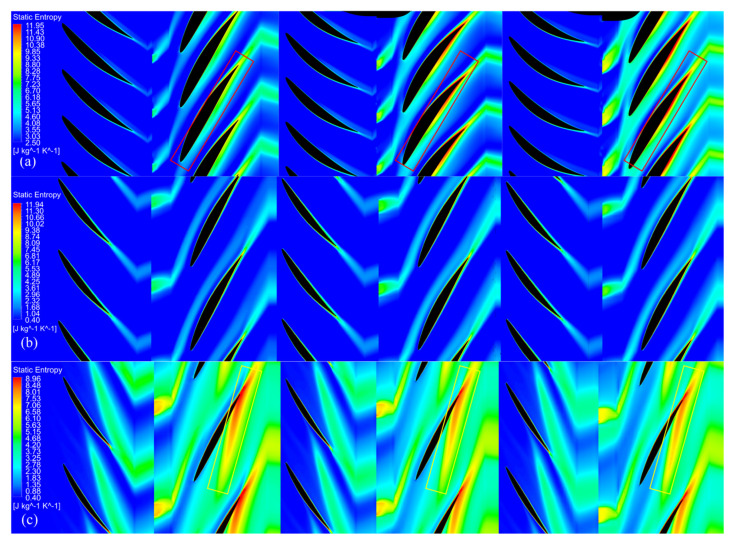
Comparison of entropy production at design points. From left to right: a = 0.3, 0.75, and 1.2, respectively, at (**a**) 3% of the blade span, (**b**) mid-span, and (**c**) 94.5% of the blade span.

**Figure 15:**
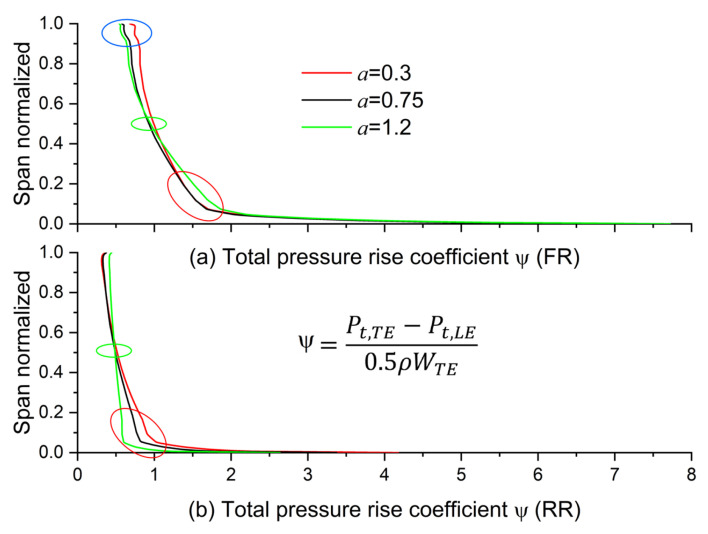
Effect of exponent on the load of (**a**) the FR and (**b**) the RR along the spanwise direction (*x* = 0.8).

Furthermore, the entropy production was investigated at three specific positions along the blade span:

At 3% of the blade span, the wake from upstream deflected to the RR blade and merged into the boundary layer of its pressure surface, which led to an increment of entropy production near the pressure surface. Thus, the boundary layer and wake were thickened, as the red rectangles illustrate in Figure 14a. However, when *a* was around 0.3, reduced FR load (as the red circle shows in Figure 15a) diluted its wake velocity and weakened its effect on the boundary layer near the RR blade root. With that, the load of RR could be increased, as illustrated in the red circles in Figure 15b, to generate a reasonable velocity distribution in Figure 7b, which led to less loss generation, as shown in Figure 13b.

At mid-span, as the green circles illustrate in Figure 15, the load variation is relatively tiny with different *a*. Therefore, the variation in entropy production was subtle in different cases in Figure 14b. In addition, the RR blade boundary layer was thinned with the reduced entropy production mentioned above compared to 3% of the blade span.

At 94.5% of the blade span, high entropy flow emerged from the FR blade’s suction side boundary layer at around the maximum profile thickness. Then, the flow developed and expanded in the FR channel and merged with the wake generated from the adjacent blade, as illustrated in Figure 14c. This mixed high entropy flow deflected to the RR blade and further developed and expanded in the RR channel. Finally, they slammed to the end of the RR blade’s pressure side, which caused a considerable loss. When *a* was around 0.3, an increased FR load (as the blue circles illustrate in Figure 15a) slightly intensified the entropy production in its wake, as shown in the yellow rectangles in Figure 14c. Consequently, the entropy production in the RR channel increased.

## 5. Conclusions

The flow behavior among the CRF cascades is complex. This article aims to investigate the regularity between the behavior and the incurred loss. Therefore, the blade design program was established to help explore the regularity under different velocity distributions. The regularity was obtained tentatively by analyzing the loss distribution and entropy production of established CRFs:

- The efficiency of the entire stage rises with increased *x* until *x* equals around 0.8 and then starts to reduce. The variation tendency of η over *a* is similar under different values of *x*: η is varied in a *Λ*-shaped curve for the front stage, a √-shaped curve for the rear stage, and an M-shaped curve for the entire stage.
- The entropy production of the front stage has a significant influence on the performance of the rear stage. Therefore, to reduce the generated loss near the annulus, the FR blade’s load allocation around the tip and root should be decreased to weaken the development of tip leakage flow and blade wake to the rear stage.
- Load allocation of the RR blade root should be increased rather than decreased to improve the performance near the annulus. Matched with the reduced velocity from the front stage, the velocity components at the TE of the RR blades decelerate further to comply with the development of the boundary layer near the annulus.
- Compared with other combinations, the optimal configuration behaves better than the others under off-design conditions. This configuration is especially superior considering the entropy production near the stall point. The development of high entropy flow near the annulus and blade surfaces can be significantly inhibited.

## Figures and Tables

**Table 1:** Design specifications.

Characteristics	Value
Tip diameter (mm)	602
Hub/tip ratio	0.598
Tip clearance (mm)	1.5
Rotational speed (rpm)	2950
Blade number (FR/RR)	13/11
Speed ratio (FR/RR)	1/1
Mass flow rate (kg/s)	9.14
Total pressure rise (Pa)	4860
Total pressure rise ratio (FR/RR)	1/1

**Table 2:** Turning angles at the RR blade root.

Characteristics	Values						
Exponent *a*	0.2	0.25	0.3	···	1.2	1.3	1.35
β_LE_ (degree)	67.9	67.8	67.7	···	65.9	65.8	65.7
β_TE_ (degree)	68.6	60.4	57.8	···	55.4	57.8	68.0
Δβ (degree)	−0.7	7.4	9.9	···	10.5	8	−2.3

**Table 3:** Grid sensitivity analysis for the validation case.

FR Surrounding Nodes	RR Surrounding Nodes	Total Pressure Rise (Pa)	Relative Change Rate (%)	Total Pressure Efficiency	Relative Change Rate (%)
264,082	205,625	4357	-	0.8806	-
361,907	313,050	4379	0.00515	0.8846	0.00449
476,752	365,384	4394	0.00328	0.8865	0.00223
704,030	533,352	4396	0.00062	0.8875	0.00112
957,263	668,519	4398	0.00041	0.8885	0.00111

**Table 4:** Blade profile data of test fan (*a* = 0.5, *x* = 0.8).

	FR Blade			RR Blade		
	Hub	Mid	Tip	Hub	Mid	Tip
Inlet relative flow angle (degree)	52.80	61.15	65.59	67.12	68.30	69.18
Outlet relative flow angle (degree)	29.24	48.18	55.18	51.93	60.62	64.80
Solidity	1.5	1.06	1.00	1.5	1.14	1.00
Incidence angle (degree)	−0.98	−1.60	0.16	−1.24	0.72	3.97
Camber angle (degree)	31.38	21.07	19.19	25.30	11.93	5.68
Stagger angle (degree)	51.91	37.78	34.17	34.29	28.38	27.63
Length of the mean camber line (mm)	195.75	135.34	145.48	231.34	185.20	171.93
Blade maximum thickness ratio	0.1	0.08	0.06	0.1	0.08	0.06

**Table 5:** Comparison of results at design point.

Characteristics	Design Values	CFD Results	Test Values
Mass flow rate (kg/m^3^)	9.14	8.73	8.79
Entire stage total pressure rise (Pa)	4282	4394	4219
FR total pressure rise (Pa)	2202	2261	-
RR total pressure rise (Pa)	2075	2132	-
Flow efficiency (vs. design) (%)	-	0.955	-
Entire stage total pressure efficiency (%)	0.881	0.887	0.839
FR total pressure efficiency (%)	0.906	0.913	-
RR total pressure efficiency (%)	0.854	0.860	-

**Table 6:** Levels of exponent and length ratio.

Characteristics	Levels
Exponent *a*	0.25, 0.3, 0.4, 0.5, 0.6, 0.75, 0.8, 0.9, 1, 1.1, 1.2, 1.3
Length ratio *x*	0.55, 0.6, 0.7, 0.75, 0.8, 0.95

## Data Availability

Not applicable.

## Abbreviations

**Abbreviations**	**Nomenclature**
*a*	Exponent
*C*	absolute velocity, m/s
$\bar{d}$	tip–hub ratio
*h*	enthalpy, J
*I*	Integral
*l*	length of the mean chamber line at the mid-span of blades, m
*N*	power, W
*P*	pressure, Pa
*r*	radius, m
*s*	entropy, J/K
*T*	temperature, K
*W*	relative velocity, m/s
*ρ*	density, kg/m^3^
Δ	increment
*η*	Efficiency
Ψ	total pressure rise coefficient
*ω*	angular speed, rpm
**Subscripts**	**Nomenclature**
Atm	standard atmosphere
CRF	contra-rotating fan
ES	entire stage
*e*	motor
FR	front rotor
LE	leading edge
*m*	mid-span of the blade
RR	rear rotor
*r*	rotor
*rel*	relative
*s*	static
TE	trailing edge
*t*	total (stagnation)
*u*	tangential velocity component
*z*	axial velocity component
1,2	inlet, outlet of the front rotor
3,4	inlet, outlet of the rear rotor

## References

[1] Lengyel-Kampmann T., Bischoff A., Meyer R., Nicke E. Design of an economical counter rotating fan: Comparison of the calculated and measured steady and unsteady results. Proceedings of the Turbo Expo: Power for Land, Sea, and Air.

[2] McKay R.S., Kingan M.J., Go S.T., Jung R. (2021). Experimental and analytical investigation of contra-rotating multi-rotor UAV propeller noise. Appl. Acoust..

[3] Luan H., Weng L., Luan Y. (2018). Numerical simulation of unsteady aerodynamic interactions of contra-rotating axial fan. PLoS ONE.

[4] Gao G., You Q., Kou Z., Zhang X., Gao X. (2022). Simulation of the Influence of Wing Angle Blades on the Performance of Counter-Rotating Axial Fan. Appl. Sci..

[5] Bandopadhyay T., Mistry C.S. (2022). Effects of Total Pressure Distribution on Performance of Small-Size Counter-Rotating Axial-Flow Fan Stage for Electrical Propulsion. ASME Open J. Eng..

[6] Ding Y., Wang J., Jiang B., Li Z., Xiao Q., Wu L., Xie B. (2022). Multi-Objective Optimization for the Radial Bending and Twisting Law of Axial Fan Blades. Processes.

[7] Pan T., Shi K., Lu H., Li Z., Zhang J. (2022). Numerical Investigations of a Non-Uniform Stator Dihedral Design Strategy for a Boundary Layer Ingestion (BLI) Fan. Energies.

[8] Kong C., Wang M., Jin T., Liu S. (2021). An optimization on the stacking line of low-pressure axial-flow fan using the surrogate-assistant optimization method. J. Mech. Sci. Technol..

[9] Adjei R.A., Fan C., Wang W., Liu Y. (2021). Multidisciplinary Design Optimization for Performance Improvement of an Axial Flow Fan Using Free-Form Deformation. J. Turbomach. -Trans. Asme.

[10] Kim Y.-I., Lee S.-Y., Lee K.-Y., Yang S.-H., Choi Y.-S. (2020). Numerical Investigation of Performance and Flow Characteristics of a Tunnel Ventilation Axial Fan with Thickness Profile Treatments of NACA Airfoil. Energies.

[11] Nouri H., Ravelet F., Bakir F., Sarraf C., Rey R. (2012). Design and experimental validation of a ducted counter-rotating axial-flow fans system. J. Fluids Eng..

[12] Ravelet F., Bakir F., Sarraf C., Wang J. (2018). Experimental investigation on the effect of load distribution on the performances of a counter-rotating axial-flow fan. Exp. Therm. Fluid Sci..

[13] (2008). Basic Types, Sizes, Parameters, and Characteristics Curve of Fans.

[14] Wu C.H. (1952). A General Theory of Three-Dimensional Flow in Subsonic and Supersonic Turbomachines of Axial-, Radial, and Mixed-Flow Types.

[15] Farokhi S. (2014). Aircraft Propulsion.

[16] Robbins W.H., Jackson R.J., Lieblein S. (1955). Aerodynamic Design of Axial-Flow Compressors. VII-Blade-Element Flow in Annular Cascades.

[17] Howell A. (1945). Fluid dynamics of axial compressors. Proc. Inst. Mech. Eng..

[18] Menter F.R. (1994). Two-equation eddy-viscosity turbulence models for engineering applications. AIAA J..

[19] Alfonsi G. (2009). Reynolds-averaged Navier–Stokes equations for turbulence modeling. Appl. Mech. Rev..

[20] (2017). Fans—Performance Testing Using Standardized Airways.

[21] Wang W., Chu W., Zhang H., Wu Y. The effects on stability, performance, and tip leakage flow of recirculating casing treatment in a subsonic axial flow compressor. Proceedings of the Turbo Expo: Power for Land, Sea, and Air.

